# Mapping the global research landscape of ischemia–reperfusion injury in cardiac surgery and transplantation: a bibliometric analysis

**DOI:** 10.1186/s13019-026-04777-4

**Published:** 2026-10-27

**Authors:** Mario Jesús Guzmán-Ruvalcaba, Jazmín Montserrat Guzmán-Díaz, Elea Mia Sara Reischl, Jil Kristin Förster, María Natalia Alonso-Jiménez, Edgar Santos, Sameer Al-Maisary, Gabriele Romano, Rawa Arif, Gábor Szabó, Matthias Karck, Sevil Korkmaz-İçöz

**Affiliations:** 1 Department of Cardiac Surgery, Heidelberg University Hospital, Im Neuenheimer Feld 326, 69120 Heidelberg, Germany; 2 Department of Neurosurgery, Oldenburg University Hospital, Steinweg 13-17, 26122 Oldenburg, Germany; 3 Department of Cardiothoracic Surgery, Saarland University Hospital, Kirrberger Str. 100, 66424 Homburg, Germany; 4 Department of Cardiac Surgery, Halle University Hospital, Ernst-Grube-Straße 40, 06120 Halle (Saale), Germany; 5 Department of Cardiac Surgery, Augsburg University Hospital, Stenglinstraße 2, 86156 Augsburg, Germany

**Keywords:** Ischemia–reperfusion injury, Cardioprotection, Cardiac surgery, Heart transplantation, Cardiopulmonary bypass, Bibliometric analysis, Science mapping

## Abstract

**Background:**

Ischemia–reperfusion injury (IRI) is intrinsic to cardiac surgery and heart transplantation, where ischemia is inevitably followed by reperfusion that paradoxically aggravates myocardial damage. Despite decades of research, no universally accepted cardioprotective strategy exists, and the global structure of this literature has not been mapped. We aimed to characterize the scientific landscape of IRI and protective strategies in cardiac surgery and transplantation.

**Methods:**

We retrieved documents (articles, reviews and proceedings papers) from the Web of Science Core Collection. No lower date limit was applied; the corpus covers the complete publication years 1979–2025. Performance indicators and science-mapping analyses (co-authorship, keyword co-occurrence, cited-reference analysis, thematic mapping and trend topics) were computed following the Bibliometrix methodology and reported per the BIBLIO guideline.

**Results:**

The corpus spanned 1979–2025 across 905 journals and 18,312 authors, accruing 180,256 citations (mean 39.5 per document; h-index 162); 43% of documents appeared in 2015–2025. The United States and China led both production and impact, although roughly 93% of Chinese output postdates 2010; Harvard University, the Chinese Academy of Medical Sciences and Semmelweis University were the most productive institutions. By h-index, the most influential identifiable authors were Heusch, Hausenloy and Yellon. The Annals of Thoracic Surgery and the Journal of Thoracic and Cardiovascular Surgery were the leading journals, and a Bradford core of 23 journals concentrated one third of output. Cited-reference analysis identified the ischemic conditioning lineage as the dominant intellectual base. Ranking protective interventions showed that cardioplegia dominates by volume while the highest citation rates belong to recent, largely preclinical topics; thematic and trend analyses showed a shift from neutrophil- and nitric-oxide–centred inflammation toward mitochondria, regulated cell death and a more recent cluster of mesenchymal stem cells, exosomes, microRNA, melatonin, ferroptosis and donation after circulatory death.

**Conclusions:**

Research on IRI in cardiac surgery has matured into a large, internationally collaborative field whose emphasis has been shifting from mechanistic description toward translational and regenerative cardioprotection, although the clinically oriented share of the literature has remained stable since 2010. This map identifies its intellectual foundations, leading contributors and emerging frontiers, offering a structured description that may help orient future cardioprotective research.

**Supplementary Information:**

The online version contains supplementary material available at https://doi.org/10.1186/s13019-026-04777-4.

## Background

Myocardial protection has been fundamental to cardiac surgery since its inception in the early 1950s, with cardioplegic arrest remaining a cornerstone of contemporary cardiothoracic practice [1, 2]. Ischemia–reperfusion injury (IRI) refers to tissue damage that occurs when blood flow is restored after a period of ischemia. Early experimental pathology, building on canine infarction models developed in the 1950s [3], established that reperfusion itself contributes to myocardial damage: temporary coronary occlusion produced necrosis whose extent depended on the duration of ischemia [4], and myocardium reperfused after transient ischemia accumulated calcium as mitochondrial calcium-phosphate deposits, with cellular edema and sarcolemmal disruption [5].

Although reperfusion is indispensable for cell survival, it paradoxically exacerbates the ischemic insult through excessive reactive oxygen species generation, mitochondrial dysfunction, calcium overload, inflammation and activation of regulated cell-death pathways [6–11]. Alongside classical apoptosis and unregulated necrosis, three regulated modalities have become prominent in this literature, defined here following the Nomenclature Committee on Cell Death [12]. Necroptosis is a caspase-independent form of regulated cell death that proceeds through the receptor-interacting protein kinases RIPK1 and RIPK3 and produces necrotic morphology with plasma-membrane rupture and release of intracellular contents [13]; RIPK1 inhibition at reperfusion reduces infarct size and adverse remodelling in murine myocardial ischemia–reperfusion [14]. Ferroptosis is an iron-dependent modality driven by collapse of cellular antioxidant defences and consequent peroxidation of membrane phospholipids, and is morphologically, biochemically and genetically distinct from apoptosis, necrosis and autophagy [15]; iron chelation and ferrostatin-1 attenuate ischemia–reperfusion-induced cardiac injury in mice [16]. Pyroptosis is an inflammatory, gasdermin-mediated form of regulated necrosis coupled to inflammasome activation [12]. Clinically, IRI is relevant to acute myocardial infarction, stroke, organ transplantation and major cardiac surgery, with local endothelial injury and microthrombosis and systemic responses that may precipitate multi-organ dysfunction [1–3, 6–8, 17]. These interlocking mechanisms have been synthesized in comprehensive clinical and mechanistic overviews of myocardial IRI and cardioprotection [18, 19]. At the microvascular level, molecular injury to the coronary endothelium and capillary bed can progress to microvascular obstruction and the no-reflow phenomenon, first characterised experimentally by Kloner, Ganote and Jennings [20], in which myocardial perfusion fails to fully recover despite restoration of epicardial flow [21–23].

In cardiac surgery, controlled ischemia followed by reperfusion is inherent, particularly during procedures requiring cardiopulmonary bypass (CPB) and aortic cross-clamping such as coronary artery bypass grafting and valve replacement, eliciting oxidative stress, endothelial dysfunction and inflammatory activation [24–29]. The consequences extend beyond myocardial stunning to the kidneys and brain, contributing to perioperative morbidity and mortality, and are modulated by ischemic duration, protection strategy, and patient comorbidity [24, 30, 31].

A distinct scenario arises in heart transplantation, where the donor organ undergoes prolonged global ischemia during procurement, storage and transport, followed by abrupt reperfusion at implantation; this provokes a sterile inflammatory response with release of damage-associated molecular patterns, innate immune activation and cytokine release that may culminate in primary graft dysfunction, acute rejection or allograft vasculopathy [24, 25, 27, 32–41]. Compared with conventional cardiac surgery, transplantation-related IRI is generally more severe. The act of reperfusion itself is similar in the two settings, since release of the aortic cross-clamp restores coronary flow; what differs is the preceding ischemia and the degree of control retained over the conditions of reperfusion. In conventional surgery the ischemic interval is short and surgeon-determined, cardioplegia can be replenished throughout it, and the reperfusate can be modified at cross-clamp release, as in terminal warm blood cardioplegia [42]. Under standard static cold storage, by contrast, the donor heart receives a single cardioplegic dose at procurement, remains globally ischemic for hours without replenishment, and is then reperfused with the recipient’s systemic blood at systemic pressure, temperature and oxygen tension. Experimentally, limiting reperfusion pressure or oxygen tension improves functional recovery of cardiac grafts [43], but such controlled reperfusion has not become routine in clinical transplantation, and ischemic time and reperfusion injury remain recognised contributors to primary graft dysfunction [44]. Ex-vivo machine perfusion and the extracorporeal reanimation of hearts donated after circulatory death are now reintroducing control over these conditions [45, 46].

The conceptual foundations of the field were laid well before the period covered by our corpus. Systemic hypothermia was proposed as a means of protecting the heart during operation in 1950 [47], chemically induced elective cardiac arrest was introduced in 1955 [48], and selective cardiac cooling followed shortly thereafter [49]. In parallel, experimental pathology defined the injury itself: the temporal evolution of necrosis after temporary coronary occlusion [4], the no-reflow phenomenon [20], the wavefront of ischemic cell death [50], and reversible post-ischemic contractile dysfunction [51]. The observation that abrupt reoxygenation of the anoxic heart itself precipitates enzyme release, the oxygen paradox [52], and its explicit articulation as reperfusion-induced damage [53] anticipated the concept later framed as a double-edged sword [54]. Cardioplegic practice was refined in the same period into potassium-based and biochemically balanced crystalloid and blood-based solutions [55–60], whose descendants remain in contemporary use, tailored to surgical and patient profiles [61–64]. These publications predate the indexed corpus analysed here; they are cited solely to provide historical and scientific context and were not part of the bibliometric dataset. Yet the pathophysiology of IRI remains incompletely understood, and no universally accepted cardioprotective strategy exists. Although the literature continues to expand, a comprehensive mapping of its publication trends, influential contributors, collaborative networks and thematic evolution is lacking. Bibliometric analysis offers an objective means to synthesize this body of work by quantifying production, revealing intellectual and social structures and highlighting emerging frontiers.

This study aimed to provide an overview of global research on IRI in cardiac surgery and transplantation from 1979 to 2025, and specifically to (i) characterize the temporal growth of publications, (ii) identify the most productive and influential contributors, (iii) map collaborative networks, (iv) analyze thematic structures and their evolution, and (v) outline research gaps and future directions.

## Methods

### Data source and search strategy

This bibliometric review was reported in accordance with the Preliminary Guideline for Reporting Bibliometric Reviews of the Biomedical Literature (BIBLIO) [65]; a completed checklist is provided as Additional file 1. Records were retrieved from the Web of Science Core Collection (WoS-CC) and exported in BibTeX format with full metadata (authors, affiliations and addresses, abstracts, author keywords, Keywords-Plus, cited references and times-cited counts).

A topic (TS) search was executed on 10 February 2026 and refined on 25 February 2026, restricted to English-language documents. The Boolean query was: TS = ((ischemia-reperfusion injury OR I/R injury OR IRI OR “reperfusion injury”) AND (cardiac OR heart OR myocardial) AND (surg* OR transplant* OR “valve replacement” OR revascularization OR CABG OR bypass OR cardioplegia OR “myocardial protection” OR “cardiac preservation”) AND (treat* OR therap* OR drug* OR pharmacolog* OR interven* OR trial* OR mechanism* OR pathway* OR preconditioning OR postconditioning OR conditioning OR hypothermia OR antioxidant* OR anti-inflammatory* OR “stem cell*” OR exosome* OR peptide* OR protein* OR agent*)) NOT TS = (liver OR hepatic OR hepat* OR kidney OR renal OR neph* OR lung OR pulmonary OR pneum*). The search returned 4593 records; of these, 28 documents dated 2026 represented an incomplete publication year and were removed, yielding a final corpus of 4565 documents published between 1979 and 2025. The complete Web of Science export is provided as Additional file 2. All citation counts and derived indicators are a snapshot of WoS-CC as retrieved on 25 February 2026.

### Eligibility criteria and time period

Eligible documents addressed IRI in cardiac surgery or heart transplantation with an explicit focus on therapeutic or protective strategies (pharmacological interventions, mechanical protection, ischemic conditioning, cardioplegia and preservation techniques). Original research articles, review articles and peer-reviewed proceedings papers indexed by WoS-CC were retained; editorials, letters, meeting abstracts, commentaries, corrections and non-indexed materials were excluded, as were studies confined to hepatic, renal or pulmonary IRI, which were removed by the NOT clause of the query. Ischemia–reperfusion injury in other non-cardiac territories (cerebral, intestinal, limb, cutaneous) was not covered by that clause; the residual proportion of such records in the corpus is quantified in the Limitations.

No lower date limit was applied: the timespan was set to all years available in WoS-CC, and 1979 is simply the earliest year for which the query returned an indexed record. The lower boundary of the corpus is therefore a property of database indexing rather than a design decision. Topic (TS) searches interrogate title, abstract, author keywords and KeyWords Plus, and WoS-CC indexes author abstracts and author keywords systematically only from 1991 onward; only 45 of the 4565 retrieved documents (1.0%) satisfy all four Boolean blocks on the title alone. Retrieval of pre-1991 literature by a query of this form is consequently very limited, a constraint previously described for retrospective bibliometric analyses in this database [66]. The analysis therefore covers 1979–2025 as complete publication years. Records dated 2026 (*n* = 28), representing an incomplete year at the time of the search, were excluded from the corpus entirely and do not contribute to any count, table or figure.

### Data selection and harmonization

After retrieval, records were checked for duplication against Web of Science accession numbers, which were unique across all 4565 records, and titles and abstracts were screened against the eligibility criteria, with full-text consultation in ambiguous cases and disagreements resolved by consensus. Because the four concept blocks of the Boolean query had to be satisfied jointly, screening did not identify further records for exclusion; the only exclusion applied after retrieval was the removal of the 28 records dated 2026. Accession-number uniqueness does not detect works indexed more than once by the database, and three such pairs were subsequently identified (two articles indexed twice under a single DOI, and one early-access record whose final version carries a transcription error in its DOI field); their retention is addressed in the Limitations. Author names, institutions, countries and keywords were then harmonized prior to analysis.

### Bibliometric analysis

Indicators and science-mapping analyses were computed following the methodology of the Bibliometrix R package and its Biblioshiny interface [67]. To ensure full reproducibility, all metrics and figures were generated and independently cross-validated with a purpose-built implementation applying identical definitions; the complete, self-contained analysis code is provided as Additional file 3. Headline indicators reconciled exactly with direct counts from the source records. The analysis code generates the complete set of figures produced during the analysis, including those not reproduced in the manuscript.

Performance indicators included annual scientific production and the compound annual growth rate (computed over the study period 1979–2025 as the geometric mean of the year-on-year change between the first and last years; this metric is sensitive to the very low output of the earliest years and should be read together with the production curve), document-type distribution, the most productive and most cited countries, institutions, authors and journals, the h- and g-indices, and the most globally cited documents. Country attribution followed the corresponding-author convention, distinguishing single-country (SCP) and multiple-country (MCP) publications. Journal scattering was assessed by Bradford’s law [68], which describes the diminishing returns of extending a literature search across journals: when sources are ranked by output and partitioned into three zones of approximately equal article counts, a small core zone accounts for the first third, and the number of journals required to accumulate each successive third increases geometrically, in the idealized form 1 : n : n². Author keywords were normalized with a curated thesaurus harmonizing British/American spelling, hyphenation, case and genuine concept synonyms (without collapsing distinct concepts); the thesaurus is provided as Additional file 4. We constructed a keyword co-occurrence network, a thematic map based on Callon centrality and density with association-strength normalization and Louvain clustering, a trend-topic timeline (median year of term use), a ranking of cited references by the number of corpus documents citing them, used to delineate the intellectual base (we did not construct the pairwise co-citation network defined by Small [69], and this indicator should therefore be read as cited-reference frequency rather than as co-citation strength), and a three-fields (Sankey) plot linking countries, keywords, and sources. To characterise the therapeutic content of the field independently of its actors, we additionally ranked myocardial-protection interventions by publication volume and citation impact. Intervention terms were matched lexically across titles, abstracts and author keywords, and the clinically oriented proportion of each was estimated by the presence of “patient(s)”, “randomised/randomized” or “clinical trial” in the title or abstract. Both are approximations and are qualified in the Limitations.

## Results

### General information

The dataset comprised 4565 documents published between 1979 and 2025 across 905 sources and authored by 18,312 researchers. By document type, the corpus contained 3613 articles (79.1%), 689 reviews (15.1%), and 263 proceedings papers (5.8%). The documents received 180,256 citations (mean 39.5 per document); the collection reached an h-index of 162 and a g-index of 265. International co-authorship accounted for 19.9% of documents, with 6.9 co-authors per document and a collaboration index of 7.1; only 132 documents were single authored. The corpus contained 6364 distinct author keywords and 7442 Keywords-Plus terms and drew on 242,821 cited references. Fifteen documents (0.3%) are flagged as retracted publications in WoS-CC and account for 344 citations, 145 of which correspond to a single 2013 review. They were retained so that the map reflects the literature as indexed and cited rather than a retrospectively curated version of it and are identified in Additional file 5; none appears among the most cited documents in Table 5.

### Annual production and growth

**Fig. 1:**
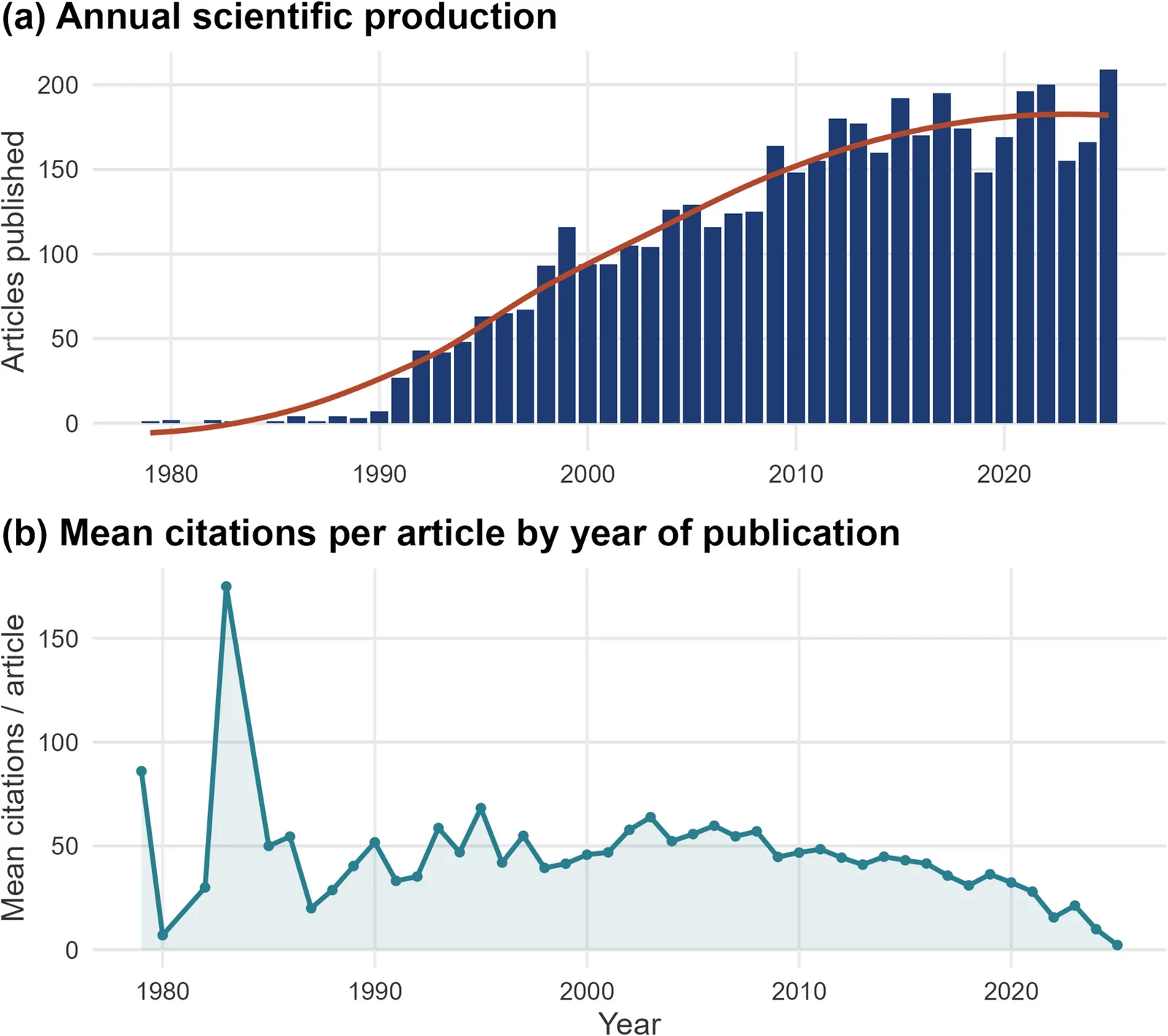
Annual scientific production and citation impact, 1979–2025. (**a**) Number of publications per year (red curve, LOESS trend). (**b**) Mean citations per article by year of publication

Scientific output grew steadily across the study period. Annual production rose from a single document in 1979 to a peak of 209 in 2025, expanding through the 1990s (571 documents) and 2000s (1181 documents) and further in the 2010s (1699 documents); 43% of all documents were published in 2015–2025. The single 1979 record [70] and the sparse output up to 1990 (26 documents, 0.6% of the corpus) reflect the indexing constraint described in Methods rather than an absence of research activity: annual counts step from 7 documents in 1990 to 27 in 1991, coinciding with the systematic indexing of abstracts and author keywords in WoS-CC. The compound annual growth rate over 1979–2025 was 12.3%, although this figure is inflated by the very low absolute output of the earliest years and is best interpreted alongside the production curve, which shows the steepest acceleration after 2000 (Fig. 1).

### Countries

**Fig. 2:**
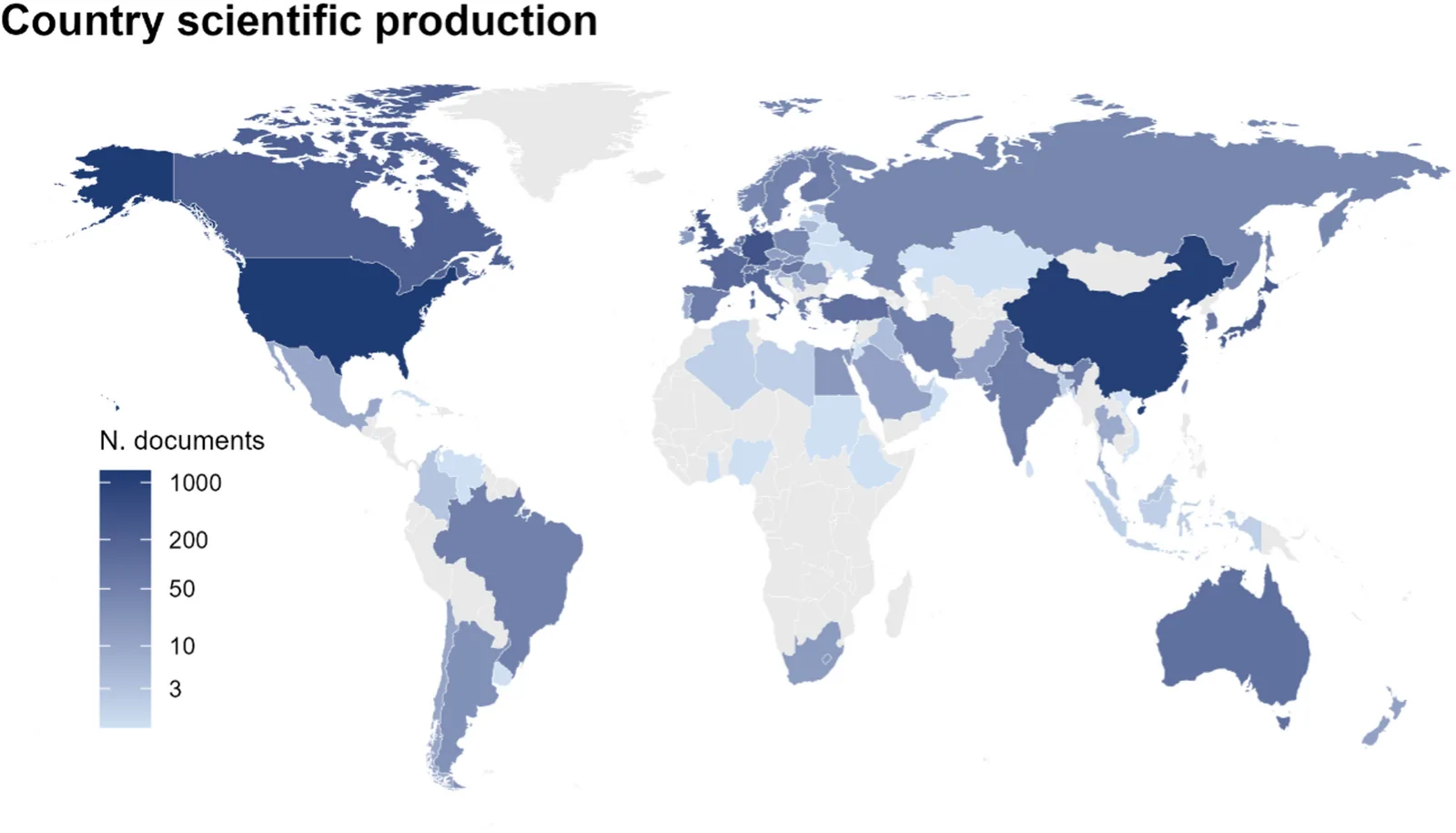
World distribution of scientific production. Countries are shaded by the number of documents with at least one affiliated author (logarithmic scale)

**Fig. 3:**
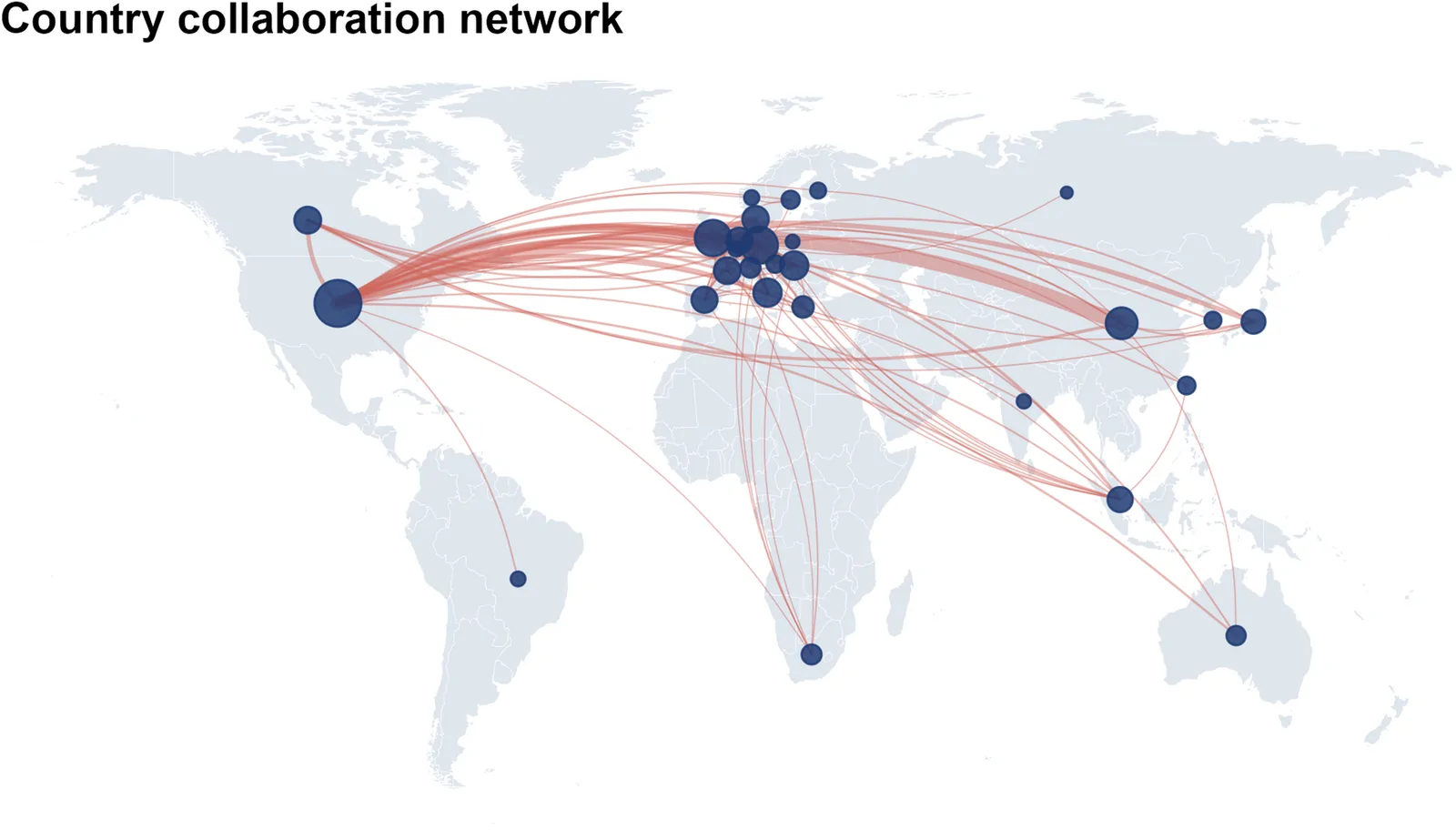
International collaboration network. Arcs link countries that co-authored at least five documents; node size is proportional to a country’s total collaboration strength

By corresponding-author country, the United States (*n* = 1130) and China (*n* = 1070) were by far the most productive, followed by Germany (*n* = 295), the United Kingdom (*n* = 250) and Japan (*n* = 221) (Table 1; complete rankings for all countries, institutions, authors, sources, cited documents, cited references and keywords are provided in Additional file 5). The same two countries led overall production counting any affiliated author (United States 1396; China 1153; Table 1), and this concentration is also visible geographically (Fig. 2). The United States (66513 citations) and China (24749) dominated impact, but the United Kingdom (16743) and Germany (13366) achieved disproportionately high citation totals relative to output (Table 1). International collaboration varied: European nations collaborated most (Germany MCP 34.9%, United Kingdom 28.4%, Italy 27.2%, France 26.0%), whereas the two leading producers were more domestic (United States 18.2%, China 11.5%) and Türkiye was almost entirely single-country (1.9%). The international collaboration network was concentrated along a North America–Europe–East Asia axis, with the United States, the United Kingdom and Germany acting as the principal connecting hubs (Fig. 3).

Aggregate counts conceal a marked difference in when this output was produced (Additional file 5, Tables S13a–b). China published 1.4% of its corresponding-author documents before 2000, 5.7% during the 2000s, 42.1% during the 2010s and 50.7% between 2020 and 2025, so that roughly 93% of its output postdates 2010. Over the same span the United States, the United Kingdom and Japan published 38–42% of their totals from 2010 onward, with output concentrated in the 1990s and 2000s. Restricting the comparison to documents published within the same window shows that the difference in citations per document is not attributable to this asymmetry: for 2000–2009 the values are 66.7 (United States) and 28.0 (China), for 2010–2014 66.5 and 28.8, and for 2015–2019 70.7 and 29.1. Only in 2020–2025 do the values converge (21.7 and 17.4), as all countries are under-cited for want of accrual time.

**Table 1:** Most productive corresponding-author countries

Rank	Country	Documents (corresponding author)	Documents (any affiliated author)	SCP	MCP	MCP %	Total citations
1	USA	1130	1396	924	206	18.2	66,513
2	China	1070	1153	947	123	11.5	24,749
3	Germany	295	398	192	103	34.9	13,366
4	United Kingdom	250	362	179	71	28.4	16,743
5	Japan	221	267	197	24	10.9	6692
6	Canada	161	229	122	39	24.2	8248
7	Italy	136	178	99	37	27.2	4217
8	France	123	160	91	32	26.0	3871
9	Türkiye	108	110	106	2	1.9	1573
10	Australia	85	108	66	19	22.4	3189
11	Netherlands	84	123	63	21	25.0	3776
12	South Korea	63	74	54	9	14.3	1726
13	Switzerland	63	81	39	24	38.1	2029
14	Finland	54	66	42	12	22.2	1236
15	India	52	62	45	7	13.5	1097

SCP refers to single-country publications, and MCP refers to multiple-country publications. Citations indicate the total number of times an item is cited within the dataset. Counts are reported without thousands of separators, in accordance with journal style.

### Institutions

Harvard University (*n* = 97) led all institutions, followed by the Chinese Academy of Medical Sciences (*n* = 63) and Semmelweis University (*n* = 62) (Table 2). Chinese universities were strongly represented (Wuhan, Shanghai Jiao Tong, Zhejiang, Fourth Military Medical), reflecting China’s rapid expansion, alongside established North American and European centres.

**Table 2:** Most productive institutions

Rank	Institution	Country	Documents
1	Harvard University	USA	97
2	Chinese Academy of Medical Sciences	China	63
3	Semmelweis University	Hungary	62
4	Wuhan University	China	52
5	University of Toronto	Canada	47
6	Huazhong University of Science & Technology	China	45
7	Shanghai Jiao Tong University	China	45
8	Zhejiang University	China	45
9	Capital Medical University	China	43
10	Fourth Military Medical University	China	43
11	Sichuan University	China	43
12	Emory University	USA	41
13	Virginia Commonwealth University	USA	41
14	Fudan University	China	40
15	Stanford University	USA	40

Counts derived from the author-address (C1) field

### Authors

Author rankings must be read with attention to name disambiguation. By raw h-index the highest-ranked signature was “Wang Y” (h = 34); however, this and other common East Asian surname–initial forms (e.g. “Zhang Y”, “Li Y”, “Liu Y”) aggregate multiple distinct individuals owing to database name homonymy and should not be interpreted as single authors (Table 3). The most influential identifiable individuals were G. Heusch (h = 32), D. J. Hausenloy (h = 32), and D. M. Yellon (h = 31), internationally recognized for foundational work on ischemic conditioning and cardioprotection. The active periods now reported in Table 3 show that the leading signatures occupy distinct eras: among identifiable individuals, Yellon DM’s indexed output is centred on 2013, Hausenloy DJ’s on 2015 and Heusch G’s on 2017, whereas earlier leaders such as Harken A and Meldrum D are centred on 1998.

**Table 3:** Most influential authors by h-index

Rank	Author	h-index	NP	Total citations	First year	Last year	Median year
1	Wang Y	34	119	5044	1999	2025	2019
2	Heusch G	32	40	6857	1998	2025	2017
3	Hausenloy DJ	32	41	5828	2006	2023	2015
4	Yellon DM	31	37	5177	2006	2025	2013
5	Zhang Y	28	88	2435	1997	2025	2020
6	Liu Y	27	75	2232	2004	2025	2020
7	Li Y	27	88	2220	1996	2025	2021
8	Li J	25	74	2703	1997	2025	2017
9	Zhang J	23	62	1936	2000	2025	2021
10	Harken A	23	31	1662	1996	2002	1998
11	Wang J	22	59	1968	2004	2025	2021
12	Meng X	22	31	1670	1996	2025	1999
13	Meldrum D	22	30	1634	1996	2006	1998
14	Zhang H	22	51	1395	1996	2025	2018
15	Zhang L	21	48	2166	2001	2025	2017

NP number of documents; TC total citations. First, last and median year refer to indexed output within the corpus. Author names are reported as indexed by Web of Science. Signatures marked by common surname–initial homonymy (e.g. Wang Y, Zhang Y, Li Y, Liu Y, Li J, Zhang J, Wang J) aggregate more than one individual, and their year columns therefore describe the pooled signature rather than any single person; see Discussion (limitations)

### Journals

The documents were dispersed across 905 journals. The most productive were the Annals of Thoracic Surgery (*n* = 149) and the Journal of Thoracic and Cardiovascular Surgery (*n* = 141), followed by Circulation (*n* = 115), the European Journal of Cardio-Thoracic Surgery (*n* = 109) and the American Journal of Physiology-Heart and Circulatory Physiology (*n* = 98) (Table 4). This mix of surgical, clinical-cardiology and basic-physiology venues illustrates the translational breadth of the field. Journal scattering showed the concentration described by Bradford’s law. Partitioning the 905 sources into three zones of approximately equal output gave a core of 23 journals (2.5% of sources) carrying 1528 documents (33.6%), a second zone of 107 journals carrying 1509 (33.1%), and an outer zone of 775 journals carrying 1517 (33.3%) of the 4554 documents with an identifiable source. The successive zone multipliers were 4.65 and 7.24 rather than the constant multiplier of the idealized model, indicating that the outer zone is more dispersed than strict geometric progression would predict, a common departure in empirical datasets [71].

**Table 4:** Most relevant sources

Rank	Source	Documents
1	Annals of Thoracic Surgery	149
2	Journal of Thoracic and Cardiovascular Surgery	141
3	Circulation	115
4	European Journal of Cardio-Thoracic Surgery	109
5	American Journal of Physiology-Heart and Circulatory Physiology	98
6	Journal of Surgical Research	71
7	Cardiovascular Research	69
8	PLoS One	68
9	International Journal of Molecular Sciences	66
10	Journal of Cardiovascular Pharmacology	65
11	Transplantation	62
12	Journal of Molecular and Cellular Cardiology	61
13	Basic Research in Cardiology	59
14	Journal of Cardiothoracic and Vascular Anesthesia	53
15	International Journal of Cardiology	48

### Most cited documents and intellectual base

The ten most globally cited documents (Table 5) span the discovery papers of ischemic preconditioning and postconditioning, landmark reviews of myocardial reperfusion injury, and remote-conditioning trials. Ranking of cited references by citing-document frequency revealed a coherent intellectual base dominated by the ischemic conditioning lineage: Murry, Jennings and Reimer’s description of ischemic preconditioning was the most frequently cited reference (481 citing documents), followed by Zhao and colleagues on ischemic postconditioning and the major reviews of reperfusion injury as a therapeutic target (Table 6).

**Table 5:** Ten most globally cited documents

Rank	First author	Year	Source	TC	TC/yr
1	Zhao Z et al.	2003	American Journal of Physiology-Heart and Circulatory Physiology	1755	73.1
2	Murry CE et al.	1986	Circulation	1673	40.8
3	Yellon DM et al.	2007	New England Journal of Medicine	1614	80.7
4	Hausenloy DJ et al.	2013	Journal of Clinical Investigation	1093	78.1
5	Heusch G	2020	Nature Reviews Cardiology	966	138.0
6	Hausenloy DJ et al.	2019	Lancet	741	92.6
7	Heusch G	2013	Lancet	730	52.1
8	Marber MS et al.	1993	Circulation	726	21.4
9	Bøtker HE et al.	2010	Lancet	691	40.6
10	Marber M et al.	1995	Journal of Clinical Investigation	663	20.7

TC total citations; TC/yr citations per year since publication

**Table 6:** Most frequently cited references (intellectual base)

Rank	Cited reference	Citing documents
1	Murry CE et al., 1986, Circulation — ischemic preconditioning of the myocardium	481
2	Zhao ZQ et al., 2003, Am J Physiol Heart Circ Physiol — ischemic postconditioning	371
3	Yellon DM & Hausenloy DJ, 2007, N Engl J Med — myocardial reperfusion injury	341
4	Hausenloy DJ & Yellon DM, 2013, J Clin Invest — neglected therapeutic target	202
5	Heusch G, 2020, Nat Rev Cardiol — cardioprotection in perspective	168
6	Piper HM et al., 1998, Cardiovasc Res — the first minutes of reperfusion	133
7	Hearse DJ et al., 1973, J Mol Cell Cardiol — the oxygen paradox	116
8	Marber MS et al., 1993, Circulation — heat stress and myocardial protection	108
9	Przyklenk K et al., 1993, Circulation — remote preconditioning	104
10	Braunwald E & Kloner RA, 1985, J Clin Invest — reperfusion: a double-edged sword	95

Reference identities are reported as resolved from the Web of Science cited-reference strings; the full ranking is provided in Additional file 5

The cited-reference layer extends well beyond the publication window of the corpus. Of 242,085 dated cited-reference instances, 4,636 (1.9%) refer to work published before 1979, distributed across 2,993 distinct references and reaching back to Langendorff (1895). Among the 30 most frequently cited pre-1979 sources, 57% of citing instances correspond to conceptual foundations of ischemia–reperfusion biology and myocardial protection, Jennings et al. on necrosis after temporary coronary occlusion (1960; 52 citing documents), Kloner et al. on the no-reflow phenomenon (1974; 52), Reimer et al. on the wavefront of ischemic cell death (1977; 37), Hearse et al. on the oxygen paradox (1973; 28), Shen and Jennings on myocardial calcium accumulation after transient ischemia (1972; 17), Melrose et al. on elective cardiac arrest (1955; 17) and Bigelow et al. on hypothermia (1950; 15). The remainder correspond to enduring experimental models (Langendorff perfusion, the isolated working rat heart, heterotopic cardiac transplantation; 19% of citing instances) and to laboratory assays that remain in routine use (24%). The foundational era of myocardial protection is therefore represented in this analysis as intellectual base and methodological inheritance rather than as units of analysis (Additional file 5, Table S11).

### Protective interventions

Ranking protective procedures and methods rather than contributors gives a different ordering of the field (Additional file 5, Table S12). Cardioplegia and its variants dominate by volume (593 documents) but attract 22.9 citations per document, with a median publication year of 2005. Ischemic postconditioning (212 documents), volatile anaesthetic conditioning (205) and adenosine (174) follow. The highest citation rates belong to recent and predominantly preclinical topics: extracellular vesicles (95.6 citations per document; median year 2022), melatonin (75.6) and microRNA (70.6). Remote ischemic conditioning combines high impact (73.1 citations per document) with the highest proportion of clinically oriented records among the high-impact interventions (80.4%), whereas mesenchymal stem cells (14.2%), histidine–tryptophan–ketoglutarate preservation (15.6%) and machine perfusion (26.5%) remain largely preclinical. Volume, citation impact and clinical orientation therefore rank the field’s protective strategies in three different orders.

### Conceptual structure

**Fig. 4:**
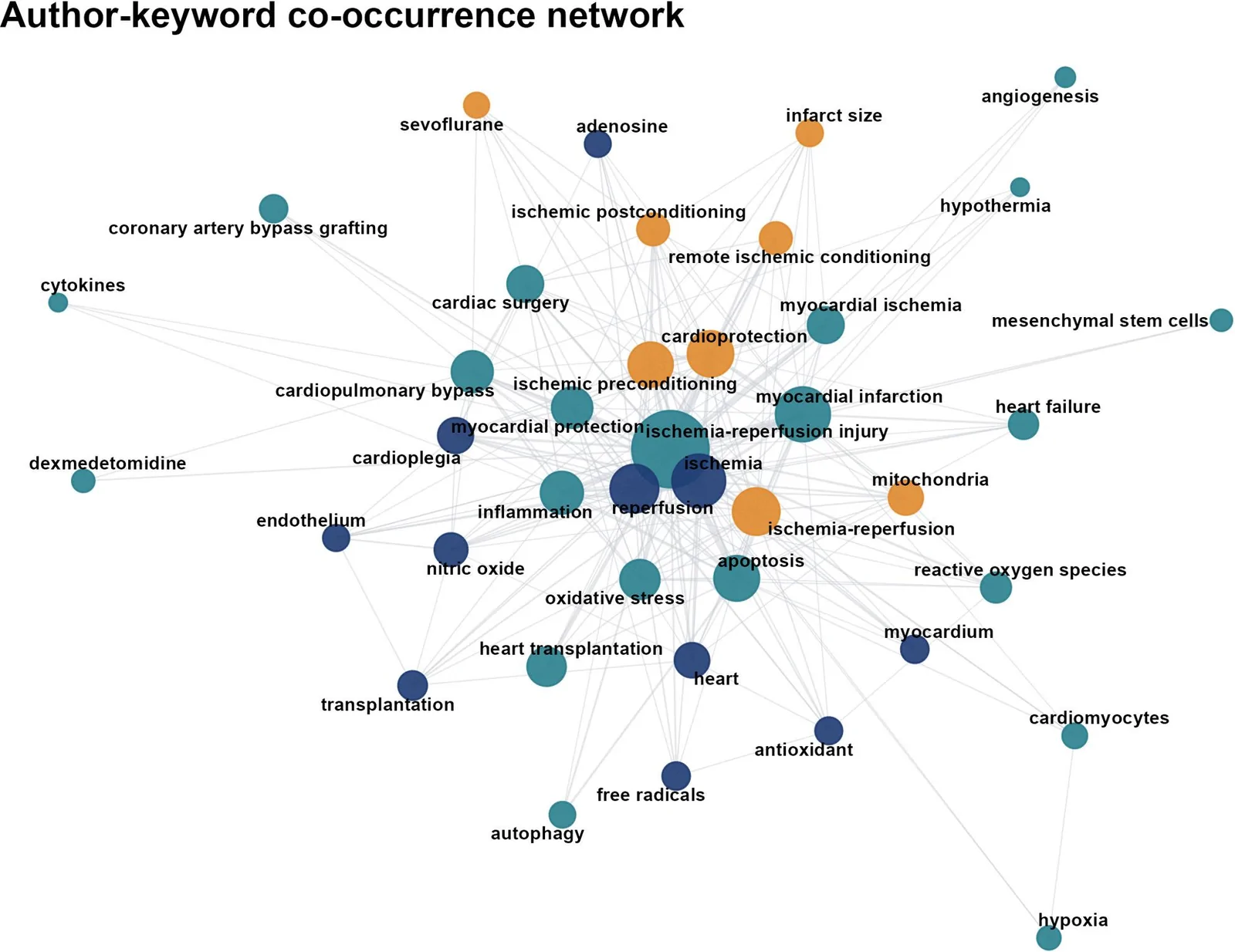
Author-keyword co-occurrence network (most frequent terms; edges with co-occurrence ≥ 6). Node size is proportional to occurrence and colour to Louvain community; layout by force-directed embedding

After thesaurus-based normalization, the most frequent author keywords were “ischemia-reperfusion injury” (*n* = 998), “myocardial infarction” (412), “ischemia” (387), “reperfusion” (295), “ischemia-reperfusion” (278), “cardioprotection” (258), “apoptosis” (248), “ischemic preconditioning” (241), “inflammation” (209) and “cardiopulmonary bypass” (195) (complete ranking in Additional file 5, Table S10). The co-occurrence network is organized around a dense core linking ischemia-reperfusion injury with ischemia, reperfusion, myocardial infarction and cardioprotection, from which mechanistic terms (oxidative stress, apoptosis, mitochondria, nitric oxide), surgical-context terms (cardiopulmonary bypass, cardioplegia, cardiac surgery, heart transplantation) and conditioning-related terms radiate (Fig. 4).

**Fig. 5:**
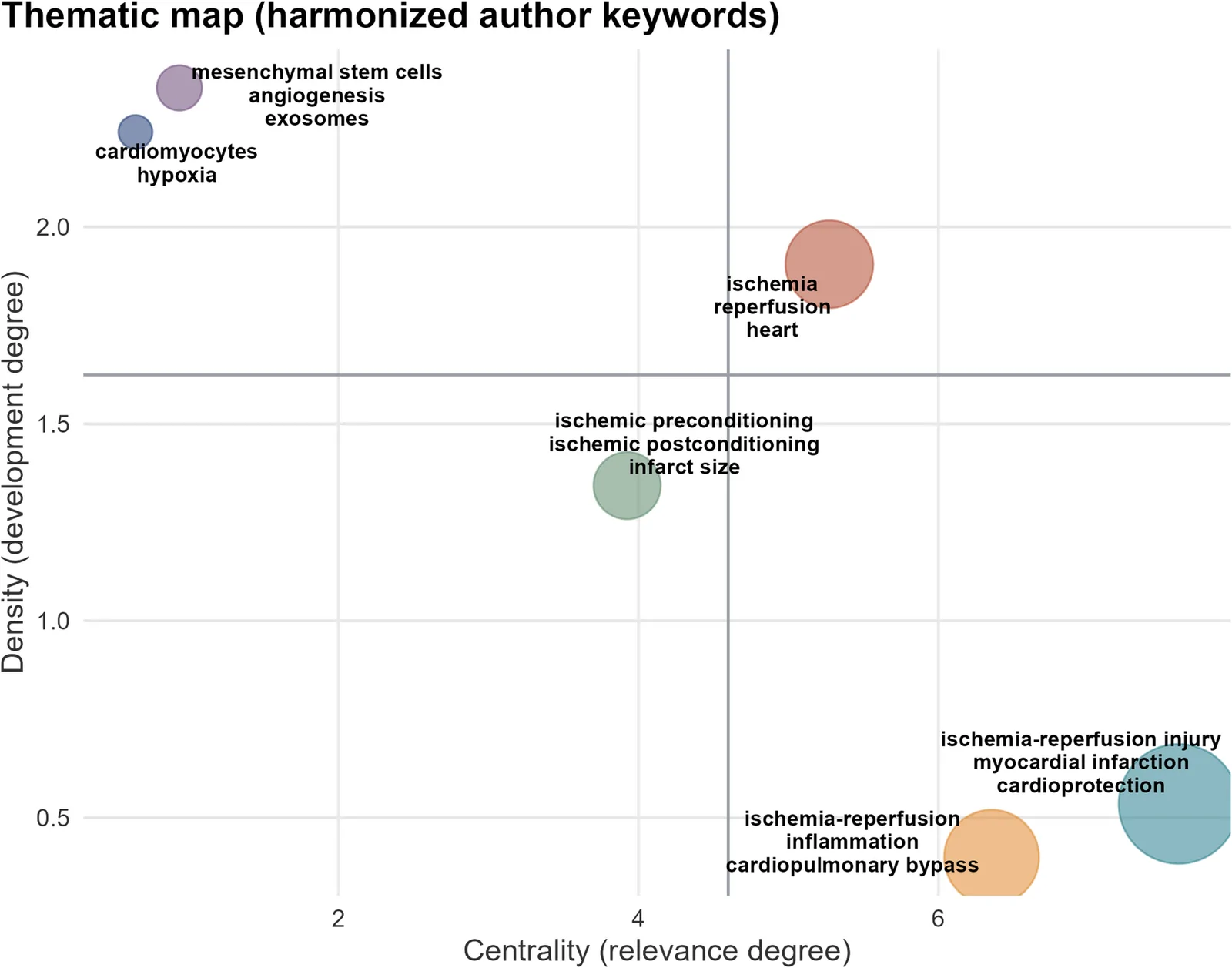
Thematic map of author keywords (Callon centrality and density; association-strength normalization; Louvain clustering). Quadrants: motor (upper-right), basic/transversal (lower-right), niche (upper-left) and emerging/declining (lower-left)

**Fig. 6:**
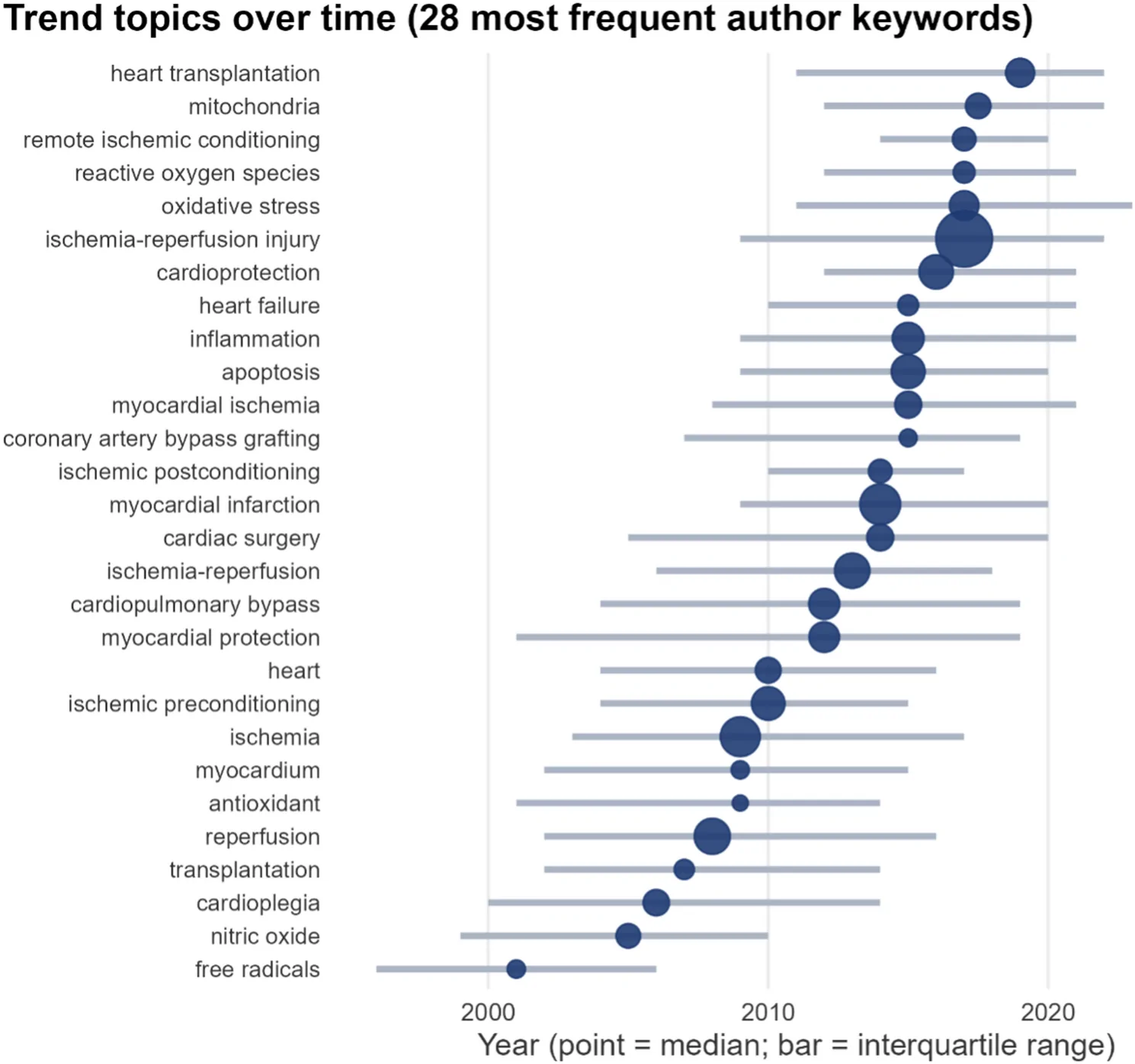
Trend topics over time (28 most frequent author keywords). The point marks the median year of use of each term and the bar its interquartile range; symbol size reflects term frequency

**Fig. 7:**
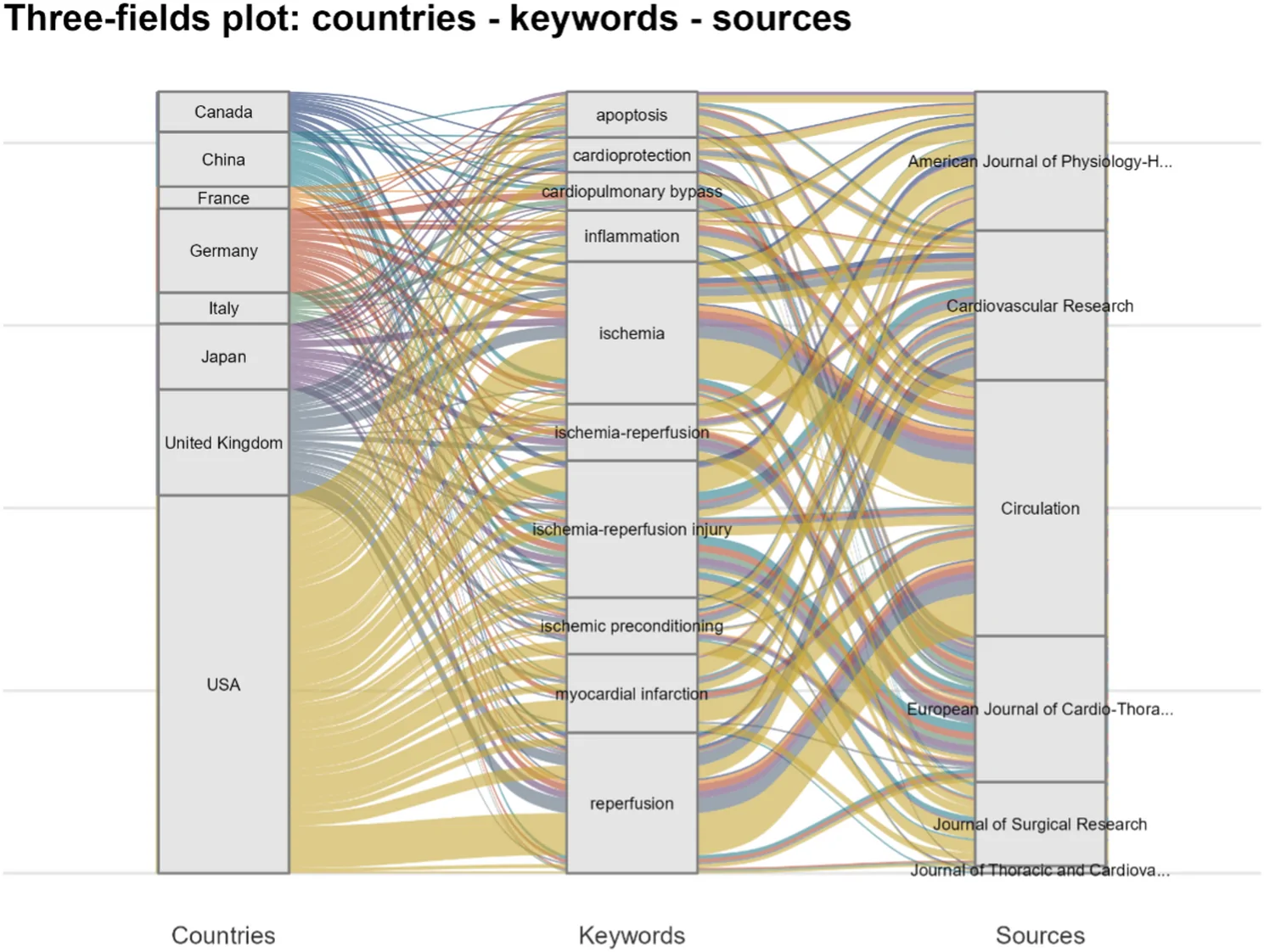
Three-fields plot (Sankey) linking corresponding-author countries (left), author keywords (centre) and source journals (right)

On the thematic map, the largest and most central clusters, ischemia-reperfusion injury with myocardial infarction and cardioprotection, and ischemia-reperfusion with inflammation and cardiopulmonary bypass, occupy the basic/transversal quadrant; an ischemia–reperfusion–heart cluster reaches the motor quadrant; the conditioning cluster (ischemic pre- and postconditioning with infarct size) lies near the centre of the map on its emerging/declining side; and mesenchymal stem cells with angiogenesis and exosomes form a niche theme (Fig. 5). The trend-topic timeline of the 28 most frequent author keywords shows this vocabulary advancing from free radicals and nitric oxide (median years 2001–2005), through cardioplegia, reperfusion and ischemic preconditioning (2006–2010), to oxidative stress, remote ischemic conditioning, mitochondria and heart transplantation (2017–2019) (Fig. 6). The most recently emerging terms fall below this frequency threshold: exosomes (median year 2022), microRNA (2020) and melatonin (2017), as reported above (Additional file 5, Table S12), together with ferroptosis, whose 39 author-keyword occurrences all postdate 2019 (median year 2024), and donation after circulatory death. The three-fields plot integrated countries, keywords and sources, confirming “ischemia-reperfusion injury” as the conceptual hub linking national outputs to specialty and mechanistic venues (Fig. 7).

## Discussion

This bibliometric review mapped 4565 documents published between 1979 and 2025 on IRI and protective strategies in cardiac surgery and transplantation. The analysis yielded three consistent patterns. First, publication growth accelerated after 2000, with 43% of all output appearing in 2015–2025, consistent with a still-expanding rather than a saturated field. Second, both production and impact were concentrated in a small set of actors: two countries (the United States and China) accounted for roughly half of all corresponding-author output, three institutions (Harvard University, the Chinese Academy of Medical Sciences and Semmelweis University) led the affiliations, and a Bradford core of 23 journals carried one third of the literature, while the highest h-indices among identifiable authors belonged to Heusch, Hausenloy and Yellon. Third, the conceptual structure shifted over time from a conditioning- and inflammation-centred base toward mitochondrial injury, regulated cell death and preservation-oriented strategies. We interpret each pattern below.

The temporal pattern reflects the scientific history of cardioprotection: the discovery of ischemic preconditioning in 1986 [72] and of ischemic postconditioning in 2003 [73], the two most frequently cited references in our dataset provided tractable, mechanistically rich models that catalyzed sustained growth, while the high citation velocity of remote-conditioning trials [74] and of integrative reviews framing reperfusion injury as a therapeutic target [75, 76] mirrors the effort to translate experimental cardioprotection into clinical benefit. The persistent prominence of CPB and inflammation as basic, transversal themes reflects the defining reality of cardiac surgery, in which IRI is an intrinsic consequence of cross-clamping and extracorporeal circulation [24, 30].

A recurring theme across the most influential literature is the gap between robust experimental cardioprotection and inconsistent clinical translation. Although ischemic conditioning reliably limits infarct size in preclinical models, large pragmatic trials such as CONDI-2/ERIC-PPCI did not demonstrate a clinical-outcome benefit of remote ischemic conditioning in patients undergoing primary percutaneous coronary intervention [77], echoing the translational challenges emphasized in contemporary perspectives [78, 79]. This persistent preclinical-to-clinical gap, rather than a lack of candidate mechanisms, frames the field’s current priorities and is consistent with the thematic shift we observed toward additive, patient-tailored and preservation-oriented strategies. The intervention-level ranking makes the translational gap concrete: the protective methods with the highest citation impact are the least clinically tested, while the most extensively studied method, cardioplegia, attracts comparatively little citation attention. Contributor rankings do not substitute for this: the authors with the highest h-indices in our corpus work predominantly on conditioning and contribute only marginally to cardioplegia research.

The geographic structure warrants careful interpretation. The United States combines the highest output with the highest impact, consistent with its dense institutional base. The near-parity between the United States and China in aggregate output rests on different temporal foundations: American output accumulated across four decades, whereas Chinese output is overwhelmingly a phenomenon of the last fifteen years. Chinese documents also show lower international co-authorship and fewer citations per document, and the latter difference persists when publication year is held constant. We report these observations without inferring from them the quality or maturity of the underlying research, which bibliometric aggregates cannot establish. The disproportionate impact of the United Kingdom and Germany and the high collaboration rates of European countries indicate that European groups act as connective hubs whose influence exceeds their raw productivity.

The conceptual evolution carries the most direct implications for future work. Over the study period, the dominant author keywords shifted from neutrophil- and nitric oxide–centred inflammation toward mitochondria, regulated cell death, and, in recent years, mesenchymal stem cells, exosomes, microRNA, melatonin, ferroptosis, and donation after circulatory death. This temporal ordering indicates a shift in research emphasis from characterizing the injury toward developing protection and toward expanding the usable donor pool, rather than a wholesale replacement of earlier themes, which remain active. The recency of the ferroptosis, non-coding-RNA and exosome clusters is consistent with the broadened pathophysiological scope (autophagy, regulated cell death and the coronary microcirculation) set out in recent syntheses [78], while the donation-after-circulatory-death and machine-perfusion terms link the basic science of IRI [6, 10] to a pressing clinical problem in transplantation. These directions should be read as hypotheses generated by the literature structure, not as evidence of clinical efficacy.

### Where the field is moving

Normalising annual output to allow for windows of unequal length shows four distinct movements over the past twenty-five years. Cardioplegia research has contracted steadily, from 20.2 documents per year in 2000–2009 to 9.2 in 2020–2025, despite cardioplegic arrest remaining in universal operative use. Conditioning research rose to 18.4 documents per year in 2015–2019 and then fell to 7.2; this contraction coincides in time with two large neutral randomised trials of remote ischemic preconditioning in cardiac surgery [80, 81], although our data establish the coincidence and not a causal link. Molecular and cellular work rose from 1.0 to 26.5 documents per year, and preservation, machine perfusion and donation after circulatory death from 2.1 to 8.8.

Set against the intervention analysis reported above, these movements describe a structural feature of the field rather than simple thematic turnover. The interventions attracting the highest citation impact are those with the smallest clinically oriented literature, and the clinically oriented proportion of the corpus has remained at 38–39% since 2010 despite substantial growth in volume. On present trends the field is generating high-attention preclinical candidates at an accelerating rate without a corresponding increase in the share of work evaluating them in patients. We report this as a property of the published literature; it is not in itself evidence about which strategies will prove clinically effective.

### Limitations

This review has limitations inherent to bibliometric methods and to its design, which we group under three headings.

*Database and retrieval.* First, the review relied on a single database (WoS-CC); although well suited to bibliometric work, the exclusion of Scopus, PubMed and other sources may omit relevant records and modestly affect counts and rankings. Second, retrieval before 1991 is constrained by database indexing rather than by our design: WoS-CC indexes abstracts and author keywords systematically only from that year, so a topic search of the present form cannot recover the 1950–1970 s myocardial-protection literature, which is represented here only through the cited-reference layer. Findings for the pre-1991 period should therefore not be read as a description of research activity in those years. Third, only English-language documents were included, introducing possible language bias.

*Corpus and metadata.* Fourth, corpus precision is imperfect in both directions: the NOT clause was applied at topic level and therefore also removed cardiac studies that mention hepatic, renal or pulmonary terms incidentally, while ischemia–reperfusion injury in other non-cardiac territories was not excluded by it, 236 records (5.2%) contain no cardiac term in their title, abstract or author keywords. Fifth, three works are represented twice in the corpus because Web of Science indexed them under separate accession numbers, which our accession-based deduplication could not detect; recomputing the principal indicators without these three records changes the document count from 4565 to 4562 and the mean citation rate from 39.49 to 39.51, and leaves the h-index (162), g-index (265) and source count (905) unchanged, so we report the corpus as retrieved. Sixth, 15 retracted documents were retained, as described in Results. Seventh, author-level productivity is affected by surname–initial homonymy: common East Asian name forms (e.g. “Wang Y”, “Zhang Y”) are collapsed by the database and conflate multiple individuals, so author prominence was interpreted primarily through the h-index and recognizable field leaders rather than raw counts. Eighth, keyword-based analyses (the co-occurrence network, thematic map, trend topics and three-fields plot) rest on author-supplied keywords, which are present in 3441 of 4565 documents (75.4%); Keywords-Plus terms were not used in these maps, and despite careful thesaurus curation, residual indexing heterogeneity may influence the fine structure of the maps.

*Analytical scope.* Ninth, no formal quality or risk-of-bias assessment of individual studies was performed; citation counts reflect attention and influence, not methodological quality. Tenth, because the analysis is restricted to complete years (1979–2025) and citation counts were captured at a single time point, citation-based indicators favor older documents and recent work is necessarily under-cited; moreover, because national outputs are distributed very differently across the study period, aggregate citation comparisons between countries conflate research characteristics with publication timing, we therefore report within-window comparisons alongside the aggregates, but neither should be read as an indicator of research quality. Eleventh, the intellectual base was delineated by ranking cited references by citing-document frequency; no pairwise co-citation network was constructed, so the relational structure among foundational works is not resolved here, and string-based reference matching may split variants of a single reference. Twelfth, the intervention ranking rests on lexical term matching, which over-counts incidental mentions and under-counts synonyms not captured by our patterns, and on a keyword-based proxy for clinical orientation that does not distinguish trials from clinical reviews; it should be read as a description of where publication and citation attention has accumulated, not as an assessment of clinical efficacy. The principal patterns reported here were nonetheless cross-validated against direct counts from the source records.

## Conclusions

Across four decades, research on ischemia–reperfusion injury in cardiac surgery and transplantation has grown into a substantial and internationally collaborative literature whose intellectual base, as shown by the ranking of cited references, is the ischemic conditioning lineage, itself built on the myocardial-protection landmarks of the 1950–1970 s. The keyword and trend analyses indicate a measurable shift over time from conditioning- and inflammation-centred work toward mitochondrial injury, regulated cell death (including ferroptosis), non-coding-RNA and exosome signalling, and donation-after-circulatory-death preservation. Read together, these patterns suggest that the field is moving from describing injury toward engineering protection, although the persistent preclinical-to-clinical translation gap means this reorientation has yet to yield a broadly effective clinical therapy. The clearest structural signal is the widening distance between where citation attention accumulates and where clinical evaluation occurs. For investigators, the map points to concrete priorities: the most-cited intellectual lineages worth building on, the most rapidly emerging keyword clusters worth testing in surgical and transplant settings, and the international hubs through which collaboration is most likely to be productive. These findings may therefore help prioritize future searches, study designs and collaborations rather than serve as a definitive account of the field.

## Supplementary Information

Supplementary Material 1

Supplementary Material 2

Supplementary Material 3

Supplementary Material 4

Supplementary Material 5

## Data Availability

All data analyzed during this study were obtained from the Web of Science Core Collection using the search strategy reported in Methods and are available to subscribers of that database. The complete, self-contained analysis code that reproduces all tables and figures is provided as Additional file 1. Supplementary tables are provided as Additional file 2 (machine-readable spreadsheet). The processed dataset is available from the corresponding author on reasonable request.

## Abbreviations

IRI: Ischemia–reperfusion injury
CPB: Cardiopulmonary bypass
CABG: Coronary artery bypass grafting
ROS: Reactive oxygen species
SCP: Single-country publications
MCP: Multiple-country publications
NP: Number of documents (publications)
TC: Total citations
WoS-CC: Web of Science Core Collection
BIBLIO: Guideline for Reporting Bibliometric Reviews of the Biomedical Literature
DAMPs: Damage-associated molecular patterns
