# Supplementary material for: Mapping the global research landscape of ischemia–reperfusion injury in cardiac surgery and transplantation: a bibliometric analysis
Source: J Cardiothorac Surg. 2026 Oct 27;21:868. doi: 10.1186/s13019-026-04777-4 (PMC13726151; doi:10.1186/s13019-026-04777-4)
Supplement: Supplementary file 1 [file 13019_2026_4777_MOESM1_ESM.docx]

**Additional file 1. BIBLIO reporting checklist**

Completed Preliminary Guideline for Reporting Bibliometric Reviews of the Biomedical Literature (BIBLIO; Montazeri et al., Syst Rev 2023;12:239) for this manuscript.

| **Section** | **Item** | **Checklist item** | **Reported in** |
| --- | --- | --- | --- |
| Title | 1 | Identification — identify the report as a bibliometric review in the title | Title page |
| Title | 2 | Issues/topics — key topics under investigation and coverage of time period | Title page |
| Abstract | 3 | Structured summary (background, methods, results, conclusions) | Abstract |
| Introduction | 4 | Justification/rationale — review of existing knowledge and epidemiological information | Introduction |
| Introduction | 5 | Objectives — statement of the objective(s) or question(s) | Introduction (final paragraph) |
| Methods | 6 | Search engines (data sources) | Data source and search strategy |
| Methods | 7 | Search strategy — keywords, date of search, language, document type | Data source and search strategy |
| Methods | 8 | Time period covered and justification | Data source and search strategy; Eligibility criteria and time period |
| Methods | 9 | Eligibility criteria — inclusion/exclusion, language, design, publication type, period | Eligibility criteria and time period |
| Methods | 10 | Data refinement (data selection procedure) — deduplication and screening | Data selection and harmonization |
| Methods | 11 | Quality assessment (optional) | Data selection and harmonization; Limitations |
| Methods | 12 | Data synthesis — methods for summarizing, handling, analyzing data | Bibliometric analysis |
| Results | 13 | Descriptive findings (statistics) — counts, years, document types, countries, impact | Results — General information; Tables 1–6 |
| Results | 14 | Schematic maps and trends — citations, journals, authors, time trends, emerging literature | Figures 1–7 |
| Results | 15 | Tabulation and summarizing the findings; historical view of first publication | Results (Tables 1–6); General information |
| Results | 16 | Synthesis of findings — identify gaps; propose model/hypothesis | Results — Conceptual structure; Discussion |
| Discussion | 17 | Summary of evidence — main findings in accessible terms | Discussion |
| Discussion | 18 | Interpretation consistent with results | Discussion |
| Discussion | 19 | Strengths and limitations | Limitations |
| Discussion | 20 | Conclusion(s) — general interpretation with respect to objectives and implications | Conclusions |
